# Post mortem laboratory detection of *Helicobacter* spp. in swine

**DOI:** 10.1007/s11259-025-10961-z

**Published:** 2025-11-01

**Authors:** Zuzana Krepelková, Viera Karaffová, Katarína Bárdová, Sandra Andrašková, Jaroslav Novotný

**Affiliations:** 1 Clinic of Swine, University of Veterinary Medicine and Pharmacy, Komenského 73, Košice, 04181 Slovakia; 2 Department of the Morphological Disciplines, University of Veterinary Medicine and Pharmacy, Komenského 73, Košice, 04181 Slovakia

**Keywords:** Swine, Gastric lesions, *Helicobacter* spp., Impression cytology, Urease test, Real-time PCR

## Abstract

Gastric ulcers in pigs are a major health issue, reducing productivity and welfare. Colonization of the gastric mucosa by *Helicobacter* spp., particularly *Helicobacter suis*, is considered a potential etiological factor. This study evaluated the prevalence of gastric lesions and compared three diagnostic methods —impression cytology, urease test, and real-time polymerase chain reaction (Real-Time PCR) for detecting *Helicobacter* spp. in pigs. *Post mortem* samples were collected from 137 slaughtered pigs, with three mucosal samples per stomach (fundus, pylorus, pars oesophagea), totaling 411. A subset of 20 samples, representing all stomach regions and diverse cytology/urease outcomes, was analyzed by Real-Time PCR targeting *H. suis* and *H. pylori* (ureA and 16 S rRNA genes). Macroscopic evaluation frequently revealed ulcerative or pre-ulcerative changes. The highest detection rates occurred in the fundic region (66% by cytology; 45% by urease test), supporting preferential colonization of glandular mucosa. Bacterial presence was also observed in the pars oesophagea, suggesting colonization varies with anatomical site and lesion severity. Real-Time PCR detected *H. suis* in 80% and *H. pylori* in 20% of samples, confirming its superior sensitivity and ability to differentiate species, which conventional tests cannot provide. Cytology enabled direct visualization of spiral-shaped bacteria, while the urease test was useful mainly at higher bacterial loads. In conclusion, combining macroscopic scoring with cytology, urease testing, and PCR allows comprehensive assessment of gastric lesions and *Helicobacter* infection. These results highlight the diagnostic advantages of molecular methods and the value of integrated approaches for surveillance and management of gastric disease in swine production.

## Introduction

Gastric ulcers in pigs represent a significant health issue that negatively impacts production parameters, deteriorates animal health, and poses a welfare risk to pig herds (Cybulski et al. 2021, 2024). These lesions most commonly occur in the pars oesophagea region, which lacks the protective layer of glandular mucosa, making it vulnerable to the effects of gastric acid and digestive enzymes (Friendship and Andrews 2024). Frequently discussed risk factors include finely ground feed, stress, irregular feed intake, and infection with *Helicobacter* bacteria, particularly *Helicobacter suis* (De Luca et al. 2021a; Luca et al. 2021b).

*Helicobacter suis* is a Gram-negative, spiral-shaped bacterium commonly colonizing the pig stomach. Its prevalence at slaughter age can reach 60–90%. Infection with this pathogen has been associated with chronic gastritis, reduced weight gain, and ulcer formation, especially in the pars oesophagea. *Helicobacter suis* was previously classified among the so-called non-Helicobacter pylori Helicobacter species, but is now recognized as a distinct species with zoonotic potential (Liang et al. 2013). Given the multifactorial nature of gastric ulceration, comprehensive stomach evaluation is essential, including macroscopic examination to visually assess mucosal integrity and detect erosions, parakeratosis, or ulcers. Macroscopic assessment serves as the first step in determining the extent and localization of lesions and guides sample collection for further laboratory analysis (Kresse et al. 2007; Straw et al. 2019). Although lesions occur most frequently in the pars oesophagea, macroscopic inspection also facilitates the identification of changes in the glandular stomach. Detection of *Helicobacter* spp. in pigs can be achieved using several laboratory methods. The urease test is a rapid biochemical method based on the ability of *Helicobacter* spp. to hydrolyze urea into ammonia, leading to alkalization and a color change in the indicator. It is simple, quick, and equipment-efficient, but its sensitivity and specificity are time-dependent (Groote et al. 2000). Cytological examination via the impression smear technique allows direct microscopic visualization of bacteria on mucosal samples. Stains such as Giemsa or 0.5% methylene blue enhance the morphological features of spiral-shaped bacteria, enabling presumptive identification of *Helicobacter* spp. with high specificity, though somewhat lower sensitivity. PCR analysis, targeting conserved regions of the 16 S rRNA gene, is among the most accurate detection methods. Previous studies have confirmed its high sensitivity (up to 100%) and specificity, making it the reference method for confirming the presence of *Helicobacter* spp. (Hoffmann 1999).

From the perspective of infection pathogenesis, the enzyme urease plays a crucial role by breaking down urea into ammonia and carbon dioxide, thereby increasing the pH in the immediate vicinity of the bacterium and enabling its survival in the acidic environment of the stomach. Urease activity is one of the main virulence factors of *Helicobacter* spp., and therefore the presence of the urease gene (e.g., *ureA*, *ureB*) is often used as a target sequence in molecular detection (Debowski et al. 2017). At the same time, it forms the basis of the urease test, which serves as a rapid, on-site diagnostic tool. Detection of the urease protein or its gene thus represents an important diagnostic and pathophysiological marker of infection. Despite several studies on *Helicobacter* spp. in pigs, data remain limited regarding the comparative diagnostic performance of cytology, urease testing, and molecular methods across different anatomical stomach regions. This study therefore addresses this knowledge gap.

The aim of our study was to compare three diagnostic methods used on gastric mucosal samples collected postmortem from pigs, focusing on their sensitivity, specificity, and practical applicability under field and laboratory conditions.

## Materials and methods

### Macroscopic examination and sample collection

**Fig. 1:**
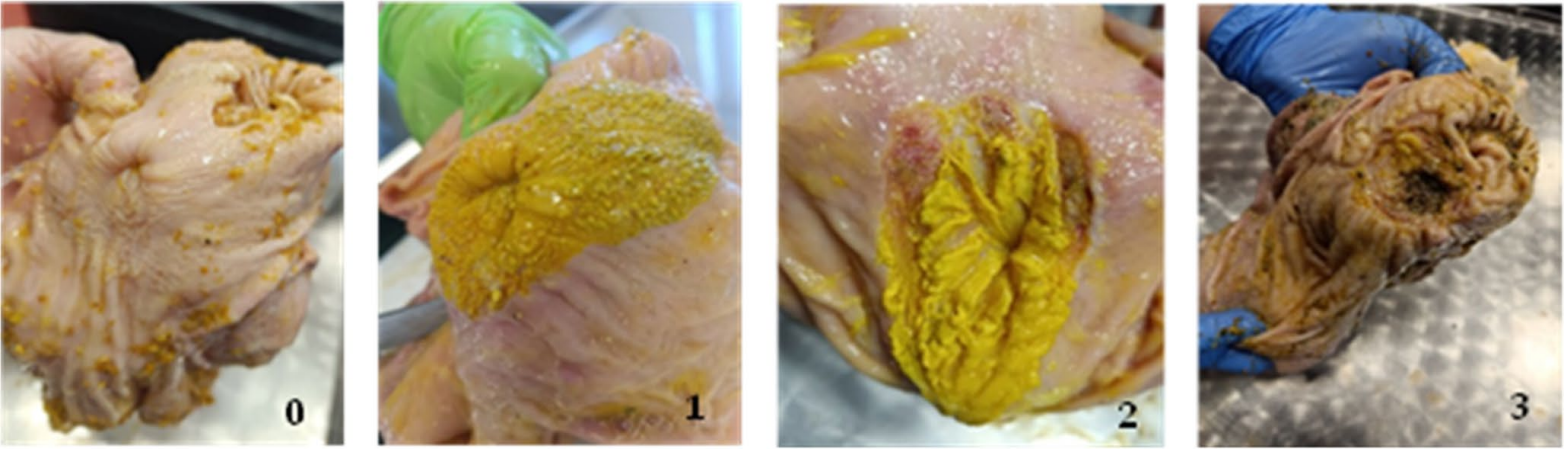
Macroscopic lesions in *pars oesophagea* (score 0–3) (own source)

Macroscopic analysis represents a fundamental and essential component of gastric ulcer diagnostics, enabling rapid assessment of the extent and severity of lesions (Ghidini et al. 2023). A total of 2,059 pig stomachs were examined at slaughterhouses during 2024–2025, shortly after slaughter, with pigs originating from various regions of Slovakia. Macroscopic evaluation of mucosal changes was always performed by the same examiner, using a modified scoring system based on the method of Robertson, which is validated for the assessment of gastric lesions in pigs (Robertson 2002). We used standardized scale (0–3): 0 – no visible mucosal damage, smooth and shiny surface; 1 – mild parakeratosis or thickening, without erosions; 2 – pronounced parakeratosis with mild erosions; 3 – presence of deep erosions, ulcerations, or scarring (Fig. 1). Of the total 2,059 stomachs examined, 137 were randomly selected for further laboratory testing (impression cytology and rapid urease test). Randomization was stratified to proportionally represent the distribution of macroscopic scores (0–3) and anatomical regions, ensuring that both healthy and ulcerated stomachs were included.

The stomachs were opened along the greater curvature (*curvatura major*) and each mucosal region was assessed, with particular attention paid to the pars oesophagea using the established scoring chart. In addition, the cardiac, fundic, and pyloric regions of the stomach were also evaluated.

Biopsy samples were collected under sterile conditions, using separate sets of instruments for each sampled stomach to prevent cross-contamination (Liang et al. 2013). Samples were transported to the laboratory within 2 h post-collection, minimizing tissue degradation and preserving sample integrity for further analyses (Klopfenstein et al. 2000).

## Laboratory methods

### Impression cytology

A total of 411 gastric mucosal samples were collected from 137 pigs, with three samples per stomach representing fundus, pylorus, and pars oesophagea. Imprints of the gastric mucosa were gently transferred onto glass slides and left to air dry. The slides were then stained with 0.5% methylene blue and examined under a light microscope for the presence of *Helicobacter spp.* (Taylor et al. 2000). This method allows direct visualization of bacteria and serves as a non-specific screening technique, complementary to urease testing and molecular diagnostics.

### Rapid urease test

For detection of urease activity of *Helicobacter* spp., the same 411 samples from the three stomach regions were examined. The samples were placed into a diagnostic medium containing urea (UREASAtest50). A positive reaction was indicated by a color change from yellow to pink/red within 1–3 h in cases of high bacterial load, or up to 24 h in cases of low bacterial load indicating the presence of *Helicobacter* spp. (Marshall et al. 1987; Thoreson et al. 1998).

This method provides a rapid, non-specific screening of *Helicobacter* presence, complementing cytological and molecular detection approaches.

### Real-time PCR detection of *Helicobacter suis* and *Helicobacter pylori*

Species-specific detection of *Helicobacter suis* and *Helicobacter pylori* was performed using Real-Time PCR. A total of 20 gastric mucosal samples were analyzed. These samples were deliberately selected due to resource limitations and to provide a purposive representation of all anatomical regions (fundus, pylorus, pars oesophagea) as well as a combination of cytology- and urease-positive, negative, and ambiguous results, allowing a meaningful comparison between diagnostic methods. Each sample was tested by four distinct Real-Time PCR assays targeting the following genes: ureA gene for *H. suis*, ureA gene for *H. pylori*, 16 S rRNA gene for *H. suis*, and 16 S rRNA gene for *H. pylori*. In total, 80 individual PCR reactions were conducted. Stomach tissue samples (20 mg) were collected in 1 mL of RNA Later solution (Qiagen, UK) and stored at − 80 °C until further processing including total RNA purification as described by Karaffová et al. (2017). Twenty samples were purposively selected from the pool of 137 examined stomachs to represent all anatomical regions (fundus, pylorus, *pars oesophagea*) and a range of cytology/urease test outcomes (positive, negative, ambiguous). This targeted approach enabled comparative assessment of diagnostic methods under different conditions.

The purity and concentration of the extracted total RNA were measured spectrophotometrically using an Implen device (Germany). The isolated RNA was subsequently reverse-transcribed into complementary DNA (cDNA) using the iScript cDNA Synthesis Kit (Bio-Rad, California, USA) with oligo(dT) primers. Relative expression of target genes including reference gene (HPRT, hypoxanthine phosphoribosyltransferase) was analyzed using Real-Time PCR on the LightCycler^®^ 480 II system (Roche, Switzerland). The reactions were carried out using the SsoAdvanced™ SYBR Green Supermix and species-specific primers (Table 1), according to the following thermal cycling program: an initial denaturation at 95 °C for 5 min, followed by 38 cycles of denaturation at 95 °C for 15 s, annealing at 60 °C for 60 s, and elongation at 72 °C for 2 min. A melting curve analysis was performed for each reaction, ranging from 65 °C to 95 °C with fluorescence readings taken every 0.5 °C. All Real-Time qPCR reactions were performed in duplicate. Quantification cycle (Cq) values of target genes were normalized against the average Cq value of a reference gene (ΔCq), and relative gene expression was calculated using the 2^–ΔCq^ method. Amplification efficiency for each target gene (including reference gene) was confirmed to be between 94% and 100% during the exponential phase of the reaction, where Cq values were determined.

**Table 1:** List of primers used in Real-Time PCR for the target gene mRNA quantification

Primer	Sequence 5’–3’	References
*H. suis* 16 s rRNA Fw	GGAGCATGTGGTTTAATTCG	In this study
*H. suis* 16 s rRNA Rev	CTTCAATGTCAAGCCTAGGT	In this study
*H. pylori* 16 s rRNA Fw	CCTCTTAGTTTGGGATAGCC	In this study
*H. pylori* 16 s rRNA Rev	AGGACATAGGCTGATCTCTT	In this study
*H. suis* urea gene Fw	AAAACAMAGGCGATCGCTCTGTA	Cortez Nunes et al. 2021
*H. suis* urea gene Rev	TTTCTTCGCCAGGTT CAAAGCG	Cortez Nunes et al. 2021
*H. pylori* urea gene Fw	AAAGAGCGT GGT TTTCATGGCG	Cortez Nunes et al. 2021
*H. pylori* urea gene Rev	GGGTTTTACCGCCACCGAATTTAA	Cortez Nunes et al. 2021

### Sequence data collection

*H. suis* and *H. pylori* 16 s rRNA gene sequences were retrieved from 3 public nucleic acid databases including GenBank (http://www.ncbi.nlm.nih.gov/), Silva comprehensive ribosomal RNA database (Silva, http://www.arb-silva.de/), and Ribosomal Database Project (RDP, http://rdp.cme.msu.edu/) in December 2024, using the following search terms: *helicobacter suis* and *helicobacter pylori*. Potential chimeric sequences were identified using Chimera Slayer and UCHIME implemented in the Mothur package (Schloss et al. 2009; Edgar et al. 2011) and subsequently removed. Database records associated with each sequence were then examined, and sequences not originating from the *Helicobacter* spp. were manually excluded.

## Results

### Macroscopic examination

**Fig. 2:**
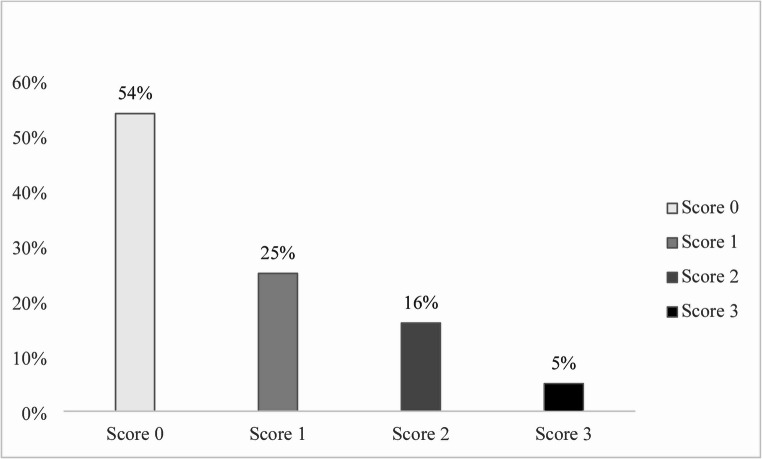
Macroscopic evaluation of the pars oesophagea mucosa

Out of all examined pig stomachs, more than half (54%; Fig. 2) showed a completely healthy *pars oesophagea* mucosa. Pre-ulcerative changes were observed in over 46% of cases, including parakeratosis (25%), erosions (16%), and gastric ulcerations (5%).

### Impression cytology

**Fig. 3:**
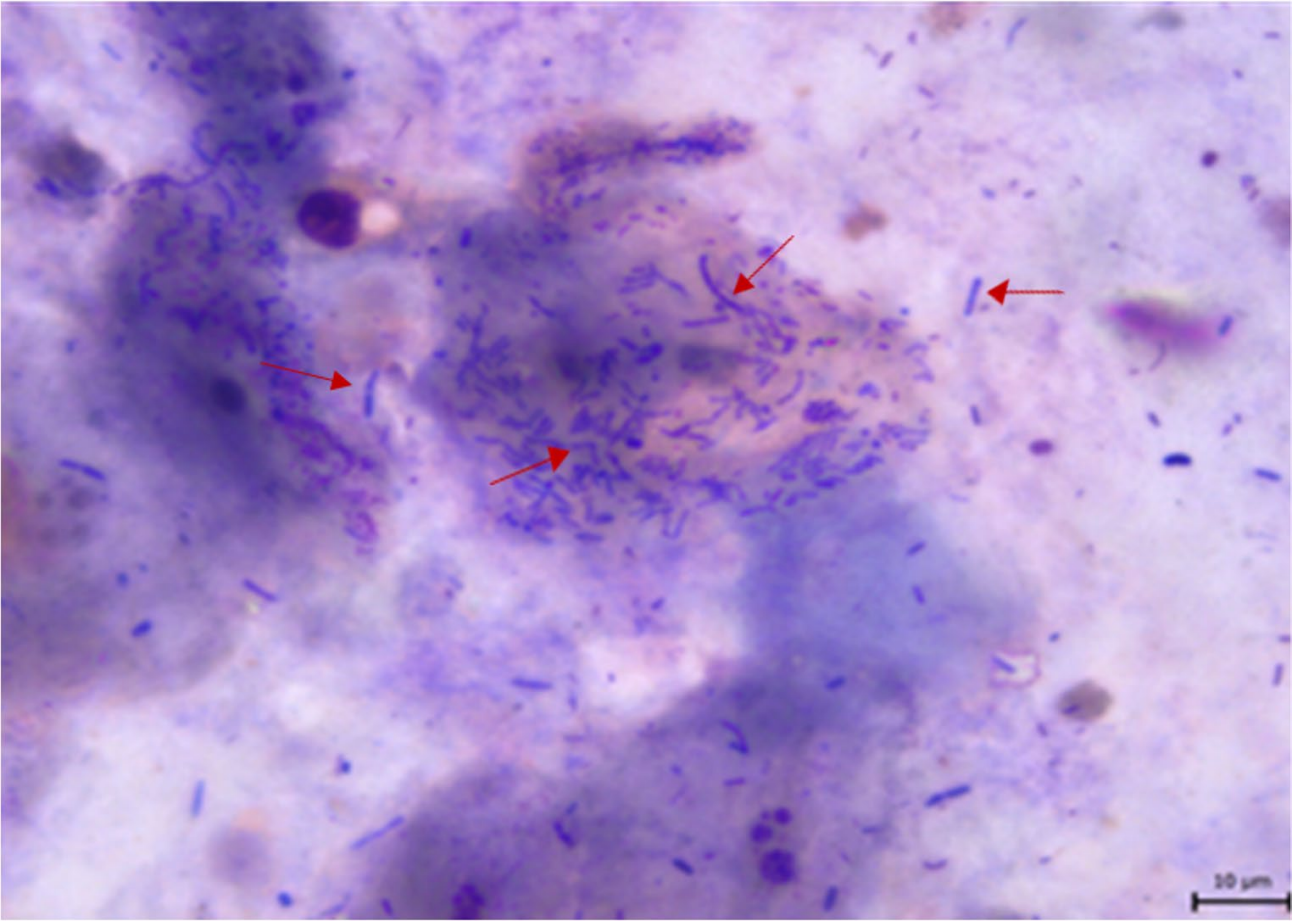
*Helicobacter* spp. in pig fundic mucosa (methylene blue staining). Legend: arrows indicate *Helicobacter spp.*

The presence of *Helicobacter* spp. observed as spiral-shaped bacteria adherent to the superficial layers of the porcine fundic gastric mucosa and visualized using methylene blue staining (Fig. 3), was confirmed with varying prevalence depending on the anatomical location.

**Fig. 4:**
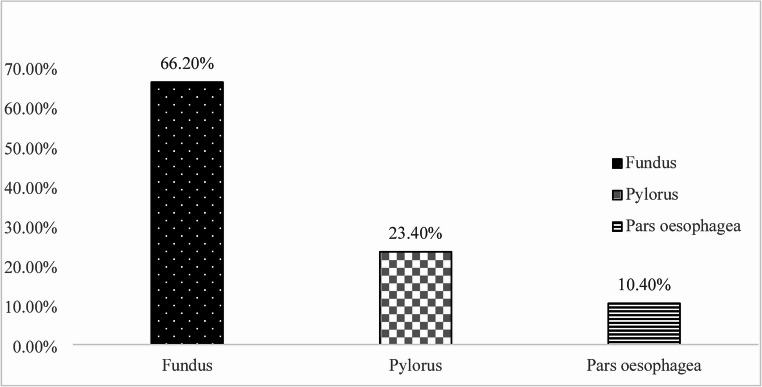
Regional distribution of *Helicobacter* spp. positivity in the porcine stomach

Among all positive *Helicobacter spp.* findings (*n* = 86), the highest proportion was recorded in the fundic region (66.2%; 57/86), followed by the pylorus (23.4%; 20/86) and pars oesophagea (10.4%; 9/86) (Fig. 4). Percentages were calculated from the total number of positive findings, with the sum normalized to 100% to clearly indicate which stomach region had the highest colonization frequency.

### Urease test

**Fig. 5:**
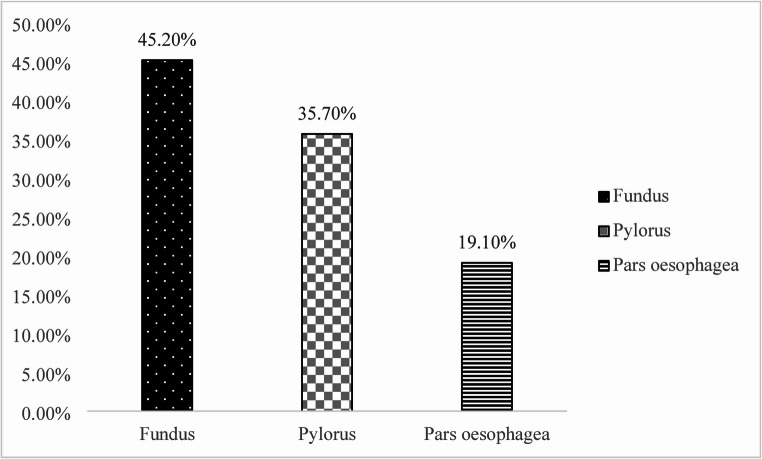
Proportion of urease-positive samples by gastric region

The urease test showed that 69% of the samples were negative (no colour change within 24 h), while 31% were positive. Among the positive samples, 9% showed a rapid reaction within 3 h most commonly in the fundic region (4.4%), followed by the pyloric (2.9%) and *pars oesophagea* (1.5%). A delayed positive reaction (within 24 h) was observed in 22% of samples, with the highest proportion again in the fundic region (9.5%), followed by the pyloric (8.0%) and *pars oesophagea* (4.4%). A total of 411 samples were examined using the urease test, of which 62 tested positive. The highest proportion of positive reactions was observed in the fundic region (45.2%; 28/62), followed by the pylorus (35.7%; 22/62) and pars oesophagea (19.1%; 12/62) (Fig. 5).

### Qualitative real-time PCR (qReal-time PCR)

The obtained results revealed that 80% samples tested positive for *H. suis*, 20% of samples were positive for *H. pylori*, and 20% of samples were negative for both species. Among the *H. suis*-positive samples, 6 originated from the *pars oesophagea* and 10 from the fundic region, supporting the known preference of this species for colonizing glandular areas of the stomach.

**Fig. 6:**
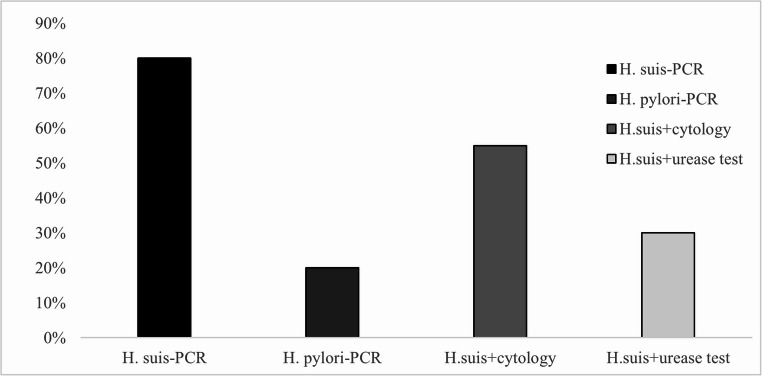
Detection of *Helicobacter* spp. by Real-Time PCR compared with cytology and urease test

These findings were compared with those obtained from non-specific diagnostic methods urease test and imprint cytology which detect the presence of *Helicobacter* spp. but do not differentiate between species. Among the 16 Real-Time PCR-positive *H. suis* cases, 6 were also positive in the urease test, while 11 showed the presence of *Helicobacter* spp. in cytological analysis (Fig. 6).

**Fig. 7:**
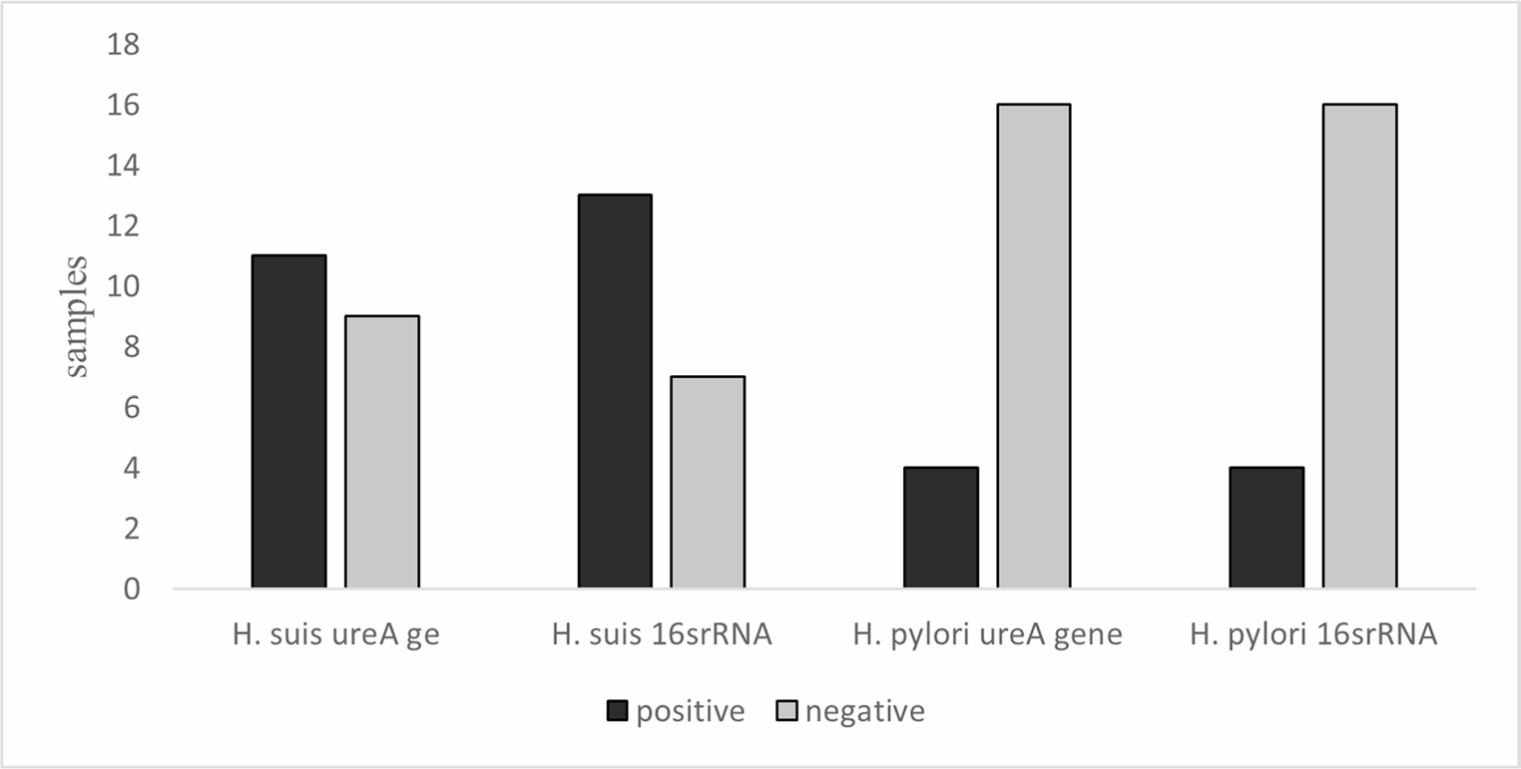
Real-Time PCR results for ureA and 16 S rRNA of *H. suis* and *H. pylori*

A detailed breakdown of the results from individual Real-Time PCR assays shows that the ureA gene of *H. suis* was detected in 11 samples, whereas the 16 S rRNA sequence of the same species was identified in 13 cases. For *H. pylori*, both Real-Time PCR assays (ureA gene and 16 S rRNA) yielded positive results in 4 samples, while 16 samples tested negative for both targets (Fig. 7). These findings suggest varying sensitivity among the individual Real-Time PCR targets and may reflect differences in amplification efficiency.

An interesting observation was the co-detection of both *H. suis* and *H. pylori* in the pyloric region in samples from three different stomachs. This indicates the potential for co-colonization of the same gastric area by different *Helicobacter* species, which may have implications for pathogenesis and diagnostic accuracy.

These findings may demonstrate the higher sensitivity of the PCR method in detecting *H. suis* and show partial concordance with the results of urease testing and cytology. Furthermore, they underscore the value of PCR method as a reference method for precise identification of *Helicobacter* species, while urease testing and cytology can serve as suitable screening tools in routine post-mortem examinations.

The detailed distribution of cytology, urease test, and PCR results for the 20 selected samples is summarized in Table 2.

**Table 2:** Comparative results of macroscopic examination, cytology, urease test, and species-specific PCR assays for *H. suis* and *H. pylori* in 20 gastric mucosal samples

Sample	Region	Macroscopic	Cytology	Urease	PCR			
					H. suis ureA	H. suis 16 S rRNA	H. pylori ureA	H. pylori 16 S rRNA
1	P. oesophagea	score 0	**–**	**–**	**–**	**+**	**–**	**–**
2	P. oesophagea	score 1	**–**	**–**	**–**	**–**	**–**	**–**
3	P. oesophagea	score 1	**+**	**–**	**+**	**+**	**–**	**–**
4	P. oesophagea	score 2	**+**	**+**	**+**	**–**	**–**	**–**
5	P. oesophagea	score 2	**+**	**–**	**+**	**+**	**–**	**–**
6	P. oesophagea	score 2	**+**	**–**	**+**	**+**	**–**	**–**
7	Fundus	healthy	**–**	**–**	**–**	**–**	**–**	**–**
8	Fundus	healthy	**–**	**–**	**–**	**–**	**–**	**–**
9	Fundus	gastritis	**+**	**+**	**+**	**+**	**–**	**–**
10	Fundus	gastritis	**+**	**–**	**+**	**+**	**–**	**–**
11	Fundus	gastritis	**+**	**–**	**+**	**–**	**–**	**–**
12	Fundus	gastritis	**–**	**–**	**–**	**+**	**–**	**–**
13	Fundus	gastritis	**+**	**+**	**+**	**+**	**–**	**–**
14	Fundus	gastritis	**+**	**–**	**+**	**–**	**–**	**–**
15	Fundus	gastritis	**–**	**–**	**+**	**+**	**–**	**–**
16	Pylorus	healthy	**–**	**–**	**–**	**–**	**–**	**–**
17	Pylorus	healthy	**+**	**–**	**+**	**–**	**+**	**–**
18	Pylorus	healthy	**–**	**–**	**–**	**+**	**–**	**+**
19	Pylorus	gastritis	**+**	**–**	**+**	**+**	**–**	**–**
20	Pylorus	gastritis	**+**	**+**	**+**	**+**	**+**	**+**

Region – anatomical site; Macroscopic – macroscopic assessment of ulcer score or gastritis/healthy; Cytology – impression cytology (*Helicobacter spp.* presence); Urease – rapid urease test; PCR assays target *ureA* or 16 S rRNA genes specific for *H. suis* or *H. pylori*; + positive; –negative.

## Discussion

Our study provides a comprehensive overview of macroscopic and microscopic changes in the pars oesophagea mucosa, as well as the occurrence of *Helicobacter* spp. in different regions of the porcine stomach. Macroscopic evaluation revealed that more than half of the animals (54%) had intact mucosa, whereas 46% showed pre-ulcerative or ulcerative lesions. These values reflect a relatively high prevalence of gastric pathology in slaughter pigs and correspond to epidemiological observations reported in comparable herds (Peralvo-Vidal et al. 2021; Ghidini et al. 2023; Cybulski et al. 2024).

Detection of *Helicobacter* spp. by cytology and urease testing confirmed an uneven bacterial distribution, with the highest colonization frequency in the fundic region (66.2% by cytology; 45.2% by urease). In contrast, the pars oesophagea exhibited markedly lower positivity rates, yet several ulcerated samples (score 2) tested PCR-positive for *H. suis* despite negative cytology and urease results. This highlights the superior sensitivity of PCR in identifying low-level or lesion-associated colonization, consistent with earlier studies (Casagrande Proietti et al. 2010; Taillieu et al. 2022).

The predominance of *H. suis* in glandular regions is well established in the literature (Liang et al. 2013; Flahou et al. 2018; De Luca et al. 2021a; Luca et al. 2021b). Our results not only support this observation but also show a clear association between the severity of pathological changes and PCR positivity. Samples with deeper erosions or ulcerations were more frequently PCR-positive, reinforcing the hypothesis that *H. suis* plays a role in the pathogenesis of gastric ulcers (Szeredi et al. 2005; Haesebrouck et al. 2009). Conversely, cytology and urease showed reduced sensitivity in mild lesions, as also reported in recent field studies (Umar et al. 2023; Pegu et al. 2024).

The heterogeneous colonization pattern was further confirmed: the fundus showed the highest detection rate, followed by the pylorus, with the pars oesophagea being the least colonized. This distribution is consistent with findings from other authors, although variations in the pyloric region have been attributed to geographic and host-related factors (Zhang et al. 2016; Kumar et al. 2019). Importantly, the occasional detection of *H. pylori* or *H. pylori*-like organisms in pigs, including in the pars oesophagea, indicates that transient colonization may occur when mucosal integrity is compromised (Oberhuber et al. 2013; Joosten et al. 2016; Queiroz et al. 2018; Michalek et al. 2020). Taillieu et al. (2024) further emphasized the role of microbiota interactions in ulcer development, suggesting a multifactorial etiology.

Physiological alterations associated with ulceration such as changes in pH, mucus viscosity, or loss of epithelial keratinization likely create microenvironments favourable for bacterial adherence. These conditions may permit colonization even in nonglandular regions, though often transiently. Because conventional cytology and urease testing can fail to detect such low-level colonization, a combined diagnostic approach is warranted. Indeed, several studies have shown that PCR identifies *H. suis* or *H. pylori* DNA where conventional tests remain negative (Casagrande Proietti et al. 2010; Taillieu et al. 2022). Comparable results were also obtained by Baele et al. (2008), Bútorová et al. (2021), and Garcia et al. 2022a).

From a practical perspective, the urease test demonstrated moderate diagnostic utility, particularly in samples with high bacterial load, and showed the best performance in the fundic region. These observations are consistent with its known time-dependent sensitivity (Groote et al. 2000; Jones et al. 2017). Cytology, while highly specific and useful for morphological confirmation (Yadav and Sagar 2022), requires careful evaluation to avoid misinterpretation due to artifacts. Thus, both methods can serve as useful screening tools in abattoir-based diagnostics, but their limitations reinforce the role of molecular methods as confirmatory tools.

Our Real-Time PCR results confirmed *H. suis* in 80% and *H. pylori* in 20% of tested samples, with positive cases originating primarily from the fundic and pars oesophagea regions. Species-specific PCR assays not only provided the highest sensitivity but also enabled differentiation between closely related species, a feature absent from conventional tests (Cardoso et al. 2022; Garcia et al. 2022a, Garcia et al. 2022b). Notably, differences between ureA- and 16 S rRNA-based assays for *H. suis* (11 vs. 13 positives) may be attributed to gene copy number or sequence conservation, while *H. pylori* assays yielded identical results (Yamazaki et al. 2011; Song et al. 2020).

The co-detection of *H. suis* and *H. pylori* in some samples suggests possible co-infections, which may influence pathogenicity, immune modulation, and clinical outcomes (Garcia et al. 2022a, b). This has implications not only for porcine health but also for zoonotic transmission, as several studies highlight occupational risks for farmers and slaughterhouse workers (Pegu et al. 2024). Consequently, our results contribute to the growing body of evidence that *H. suis* should be considered a pathogen of both veterinary and public health importance.

Taken together, these findings underline the necessity of a multimodal diagnostic approach. Macroscopic evaluation allows rapid lesion scoring, cytology and urease provide accessible on-site screening, while molecular methods remain indispensable for accurate species identification and for detecting low-level or mixed colonization. The integration of these approaches can improve surveillance and contribute to better herd health management.

Limitations of this study include the relatively small PCR-tested subset, cross-sectional design, and lack of longitudinal data, which restricts assessment of infection dynamics over time. Nevertheless, the consistent agreement of our results with published data strengthens their validity. Future studies should increase sample sizes, explore temporal colonization patterns, and investigate the role of co-infections with other gastric microorganisms. Additionally, the development of rapid, field-applicable molecular assays could facilitate routine herd-level diagnostics and early intervention.

## Conclusion

Our study provides additional insights into the prevalence and anatomical distribution of *Helicobacter* species in the stomachs of pigs, which has so far been only partially documented in the literature. The highest level of *H. suis* colonization was recorded in the fundic region, confirming its affinity for the glandular parts of the stomach. However, a detectable bacterial presence was also observed in the pars oesophagea, suggesting that their distribution may be influenced by various factors, including geographical conditions, host characteristics, and the diagnostic methods used. This variability underscores the importance of comprehensive sampling from multiple anatomical regions to more accurately determine colonization patterns. By combining macroscopic assessment, imprint cytology, urease testing, and Real-Time PCR method, we provided a comprehensive overview of the occurrence of *Helicobacter* spp. and their association with pathological changes in the gastric mucosa. While cytology and the urease test serve as useful screening tools, our results clearly demonstrate that molecular methods, particularly Real-Time PCR, are essential for precise species identification, differentiation of *H. suis* from *H. pylori*, and achieving higher sensitivity and specificity. Looking forward, our findings highlight the need for future research to develop field-applicable, rapid diagnostic tools for Helicobacter spp., which could enable earlier detection and monitoring of infection in pig populations. Larger-scale studies are also warranted to investigate co-infection dynamics, the influence of environmental and host factors on bacterial distribution, and the long-term impact on gastric health. Such studies could inform more effective prevention, management, and treatment strategies, ultimately improving animal welfare and production outcomes.

## Data Availability

No datasets were generated or analysed during the current study.
